# Does virtual reality reduce pain in pediatric patients? A systematic review

**DOI:** 10.1186/s13052-019-0757-0

**Published:** 2019-12-30

**Authors:** Anna Maria Iannicelli, Daniele Vito, Concetta Anna Dodaro, Pasquale De Matteo, Rita Nocerino, Angela Sepe, Valeria Raia

**Affiliations:** 1 Department of Translational Medical Sciences, University of Naples “Federico II”, Via Pansini 5, 80131 Naples, Italy; 2 Department of Advanced Biomedical Sciences, University of Naples Federico II, Naples, Italy; 3 Department of Internal Medicine (Metabolic and Cardiac Rehabilitation Unit), University of Naples “Federico II”, Naples, Italy

**Keywords:** Virtual reality, Pain, Pediatric, Children

## Abstract

Virtual Reality (VR) as a tool for pain reduction is the research topic of several clinical trial for Randomized Controlled Trials despite its wide use in the daily clinical practice for non- pharmacological reduction of pain in some countries. At present, there are no published reviews of VR-efficacy of pain reduction in pediatric patients. That is why we made a systematic review of the efficacy of VR as a tool for pain reduction in children and adolescents. Electronic databases and gray literature published between 2014 and 2019 were analyzed. A total of 9 studies were eligible according to the established inclusion criteria. Results show that virtual reality is a valid tool for non-pharmacological pain reduction and that this approach is to be preferred to the standard reduction techniques currently in use. However, more studies using standardized experimental methodologies are needed to provide more systematic comparison and quantitative synthesis.

## Introduction

Virtual Reality (VR) has been defined as a “relatively new tool of human-computer interactions for a human becoming an active participant in a virtual world” [1]. VR was conceived in the second half of the 90s for military exercises, but thanks to continuous technological advancement, it was soon deemed as a therapeutic tool. As a matter of fact, this new procedure was used in a cohort of soldiers who have Post-traumatic Stress Disorder (PTSD) as an alternative to exposure treatment for veterans [2].

For this reason, VR quickly became a subject of study in the whole medical-therapeutic field, presenting itself as a valid alternative to Exposure Therapy (ET) defined as Virtual Reality Exposure Therapy (VRET) [3]. One of the best-known uses of VR in the scientific literature is for treating phobias and social disorders [4]. In addition to its therapeutic use, VR can also be used for all training varieties, representing a step between theoretical and practical preparation, which was not previously feasible. VR can be realized through several tools, including personal computer screens, mobile devices and dedicated VR rooms. The most often used method for “immersion” into VR is a head-mounted visor, which can be connected to a personal computer or linked to a mobile phone. VR is not only an alternative to ET. Through its use, it is also possible to create specific environments by controlling all the elements within them. Interaction with other people, within the same virtual reality, is possible too, in case operator-patient or patient-patient are present.This review aims to introduce the use of VR as a non-pharmacological pain reduction tool in pediatric patients as a subject of a systematic review.

## Aims

To select the articles for the review, we asked 2 questions:

1. Does VR reduce pain in pediatric patients?
2. Does VR reduce pain more than standard care in pediatric patients?

## Methods

We have been searching in the following databases from January 2014 to October 2019: Embase (via the Ovid search engine), Medline (via the Ovid search engine) and the Cochrane Central register of controlled trials. We decided to exclude animal studies. The search was originally run in January 2018, in July and October 2018 and then updated in October 2019. Two investigators independently screened titles and abstracts to generate a list of articles investigating the effectiveness of any VR intervention in pediatric patients. As a second step, all authors screened the list of these articles to identify those eligible. Full articles of any potentially eligible studies were retrieved and examined.

to check against the inclusion criteria. Discrepancies were solved by further discussion with an independent investigator.

### Inclusion and exclusion criteria

We included studies involving pediatric patients aged from 0 to 18 years. We decided to include a study with a patient aged 20 years as it appeared significant for the review aim. We excluded studies with less than 3 participants because they would have been insignificant. We included studies that examined any type of VR. We excluded studies without digital data on pain perception.

**Fig. 1:**
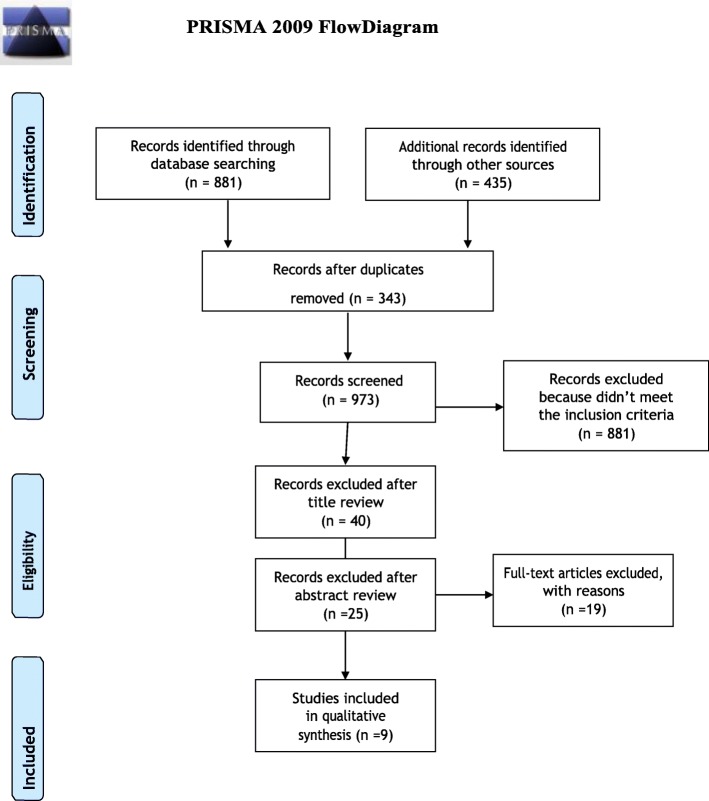
PRISMA flow diagram

We included any type of study except for literature reviews. We only included studies published in English. As shown by PRISMA Diagram (Fig. 1), the paper research started by matching the words “pain” and “virtual reality” on the electronic databases, producing a total of 1316 papers, with 343 duplicates. Therefore, we applied the following inclusion criteria. Sample size age: 0–18 years, publication date: January 2014 to October 2019. The majority of the articles were excluded because they were classified as feasibility studies or because they did not provide digital data on pain. At the same time, another part of the screened paper included a smaller part of pediatric patients with an age range > than 18 years. Additional excluded studies include articles focused on the effects of VR on anxiety and which only mention VR’s application for pain reduction. More articles were excluded because they were focused exclusively on the potential learning enhancement of VR. Therefore, at the end of the selection, there were only 9 articles published between 2014 and 2019 focused exclusively on the pediatric population and with reported digital pain data.

## Results

The search from January 2018 to March 2019 retrieved a total of 1316 papers (Fig. 1). We screened all the records and made the first selection by reading titles and then abstracts. Finally, we read the full text of the remaining 9 articles. The number of participants in each study ranged from 4 to18 years (1 study had a participant aged 20 years), while the sample size ranged from 30 to 121 subjects. Four articles were written in the United States, 1 in Canada, 1 in China, 1 in India, 1 in Syria e 1 in Australia. Four of 7 studies were published during 2018, a significant scientific year of interest; 3 articles were published in 2019, 1 paper in 2015 and 1 study in 2014.VR was used to reduce pain in the following procedures: vaso-occlusive pain episodes (VOE), inferior alveolar nerve block (IAN), immunization, pulp therapy, phlebotomy, dressing changes, burn wound care, IV placements and venipunctures. We summarized the major findings and their main characteristics (Table 1). Agrawal et al. used VR to manage vaso-occlusive pain episodes in patients with sickle cell disease (SCD). Even if one patient was 20 years old, we decided to include this study since most patients were in the pediatric age range. Pain was evaluated using the validated adolescent pediatric pain tool (APPT). Results showed the feasibility of the study and a reduction of median pain intensity: Pre-VR = 7.3; Post-VR = 3.0. In addition, the number of affected body areas decreased: pre-VR = 3.0 post-VR = 2.0 [5]. Al-Halabi et al. used VR to reduce pain in child behavior management during an inferior alveolar nerve block. Children were divided in three groups as follows: Group A (Control group), IAN administrated with basic behavior guidance techniques; Group B: IAN administrated using AV eyeglasses ‘VR box’ and wireless headphone; Group C: IAN administrated using tablet device and wireless headphones. No difference in pain level was found between the control group and the group that used Virtual Reality [6]. Chad et al. used VR to reduce pain and fear during immunization. The study also collected data on parents’ pain and perception of fear in their child while using VR. Anticipatory pain and fear were registered before the immunization, and both values decreased in 94.1% of children after the immunization with VR headset [7]. A Wong-Baker pain scale (score 0–5) was used to register the pain. On average, pain decreased by 2.57 points. Also, fear significantly decreased, and both pain and fear significantly decreased in parents’ perception too.

**Table 1:** Major findings

ID	Title	First author	Publication year	Country	Sample size	Age	Study design	Virtual reality pattern	VR application
1	Virtual reality as complementary pain therapy in hospitalized patients with sickle cell disease	Agrawal A.K.	2019	United States	31	13–20	Feasibility study	Oculus Rift VR headset	VR was used to manage pain of vaso-occlusive pain episodes (VOE) in patients with sickle cell disease (SCD).
2	Effectiveness of audio visual distraction using virtual reality eyeglasses versus tablet device in child behavioral management during inferior alveolar nerve block	Al-Halabi M.N.	2018	Syria	102	6–10	Randomized Controlled Trial	Virtual reality box (VR Box)	VR was used to reduce pain in children behavioral management during inferior alveolar nerve block.
3	Effect of virtual reality headset for pediatric fear and pain distraction during immunization	Chad R.	2018	United States	34 (17 children and 17 parents)	Children: 6–17 Parents: 32–46	Pilot study	VR Headset	VR was used to reduce pain and fear during immunization.
4	Virtual Reality for Pediatric Needle Procedural Pain: Two Randomized Clinical Trials	Chan E.	2019	Australia	252	4–11	Randomized Controlled Trial	Google Pixel XL/Google Daydream	VR was used to reduce the pain during needle-related procedures, in 2 environments: emergency department and outpatient pathology.
5	A Randomized Controlled Trial of the Use of Virtual Reality for Needle-Related Procedures in Children and Adolescents in the Emergency Department	Dumoulin S.	2019	Canada	59	8–17	Randomized Controlled Trial	eMagin z800 HMD	VR was used to reduce the pain during needle-related procedures.
6	Effects of distraction using virtual reality technology on pain perception and anxiety levels in children during pulp therapy of primary molars	Niharika P.	2018	India	40	4–8	Randomized Controlled Trial	Google VR Box and Anti-Tank Virtual Reality 3D Glasses	VR was used to reduce pain during pulp therapy in pediatric patients.
7	Effects of Virtual Reality and External Cold and Vibration on Pain in 7- to 12-Year-Old Children During Phlebotomy: A Randomized Controlled Trial	Gerçeker G.Ö.	2018	United States	121	7–12	Randomized Controlled Trial	Samsung Gear Oculus headset	VR was used to reduce pain during phlebotomy.
8	The Effect of Virtual Reality Distraction on Pain Relief During Dressing Changes in Children with Chronic Wounds on Lower Limbs	Hua Y.	2015	China	65	4–16	Prospective randomized study	Head-mount display	VR was used to reduce pain resulted from dressing changes in pediatric patients with Chronic Wounds on Lower Limbs.
9	Effect of virtual reality on adolescent pain during burn wound care	Jeffs D.	2014	United States	30	10–17	Randomized Controlled Trial	Tripod-arm device	VR was used to reduce procedural pain during burn wound care.

Chan et al. used VR to study its effect on pain perception during venipunctures and intravenous cannulation and no difference in pain between venipuncture and intravenous cannulation were reported. The study was carried out in 2 different environments: emergency department and pathology. Also, the topical local anesthetic use was high. The child-rated Faces Pain Scale-Revised was adopted to assess the pain (score 0–10). The patients underwent procedures in the emergency department experienced a reduction in pain perception of 1.78, while the patients in pathology experienced a reduction of 1.39. In addition, fewer people were required to restrain patients during the procedures. The sample size analyzed was not numerically sufficient in the statistical analysis [8].

Doumlin et al. investigated the efficacy of VR as a mode of distraction during venipunctures and intravenous cannulation, comparing it with watching television and with distraction provided by the Child Life program. The authors demonstrated that although a reduction in fear of pain was observed, no differences were found in pain intensity. Again, topical anesthetic was applied to the majority of participants before the procedures. The authors also stated that the sample size analyzed was numerically sufficient in the statistical analysis [9].

Niharika et al. used VR to reduce pain during pulp therapy in pediatric patients. The study provided 3 sessions and 2 groups (A = 20 children; B = 20 children). In the first session no children used VR. In the second session Group A used VR while Group B did not use it. In the third session Group A did not use VR while Group B did use it. Faces version of the Modified Child Dental Anxiety Scale (MCDAS[f]) Questionnaire was used to evaluate state anxiety and a Wong–Baker Faces Pain Rating Scale was used to assess pain perceived during dental procedures. Group A’s pain value in the second session was 2.56 ± 0.39; in the third session pain was 5.22 ± 0.515. In Group B pain value was 5.44 ± 0.682 in the second session; in the third session pain was 2.33 ± 0.37. In conclusion, VR significantly reduced pain in both groups. In addition, pain reduction in Group B was more intense [10]. Gerçeker et al. used VR to reduce pain during phlebotomy. Patients were randomly allocated to 3 groups (1 using VR, 1 using external cold and vibration and 1 used as control group). Results showed that no statistical difference was found between the groups using VR and the group using external cold and vibration, according to the pain scores reported by children themselves, parents, the nurse, and the researcher. Anyway, reported pain was statistically lower in groups 1 and 2 compared to group 3 [11]. Hua et al. used VR to reduce pain while changing dressing in pediatric patients with Chronic Wounds on Lower Limbs [12]. The procedure included undressing, cleaning the wound, and getting a different dress after a doctor assessed the wound. Children rated their pain before, during, and after the dressing changes with a Wong–Baker Faces (FACES) picture scale. Also, caregivers and nurses registered pre-, intra- and post-dressing change pain with the visual analogue scale (VAS) and the Face, Legs, Activity, Cry, Consolability (FLACC) pain behavior scale. The results of the Wong–Baker scale follow before dressing change: Standard Distraction = 1.63 ± 1.39, VR Distraction = 0.85 ± 1.12 during dressing change: Standard Distraction = 4.19 ± 2.12, VR distraction = 2.42 ± 1.85 after dressing change: Standard Distraction = 3.38 ± 1.48, VR distraction = 2.48 ± 1.8. Also, VAS and FLACC showed pain reduction during every step. Along with pain, time length for dressing changes was registered in both groups: 27.9 ± 6.83 min for the standard distraction group vs 22.3 ± 7.85 min for the VR distraction group. Another study used VR to reduce procedural pain during burn wound care [13]. Participants were randomly assigned to three groups: standard care, passive distraction watching a movie or VR distraction. A 100-mm line word graphic rating scale (WGRS) was used to measure the procedural pain. Participants in the VR group reported significantly less procedural pain than the passive distraction group, with a difference of 2.37 cm in the WGRS.

## Discussion

In this review we observed that no studies investigated the use of VR compared to standard cares in the pediatric population; most of them did not show the effective difference of pain perception between the use of VR and the standard care even if a lot of records were retrieved. In fact, several studies evaluated the effectiveness of VR distraction for reducing experimental pain experienced by both adults and children [14]. In the last five years no studies investigated the reduction of pain in children and no review in this field was found. Although the procedures described in the selected articles are different, the use of VR as a non-pharmacological reduction technique was observed in the procedures related to needle puncture, such as immunization, and medication management, such as dressing care. One of the studies analyzed the difference in pain detection with and without VR among patients who had taken opioids and patients who had not taken them: the former group, unlike it could be assumed, reported a lower pain reduction than the latter group [13]. However, it is not possible to carry out a significant analysis of these results since the study did not aim to analyze this difference. The studies used different VR patterns but the head-mounted was the more used. Moreover, in one study 2 groups of patients carried out the immersion at different times [10]. A group received the immersion in VR before the painful procedure was carried out and then the procedure was carried out without the VR, with double perceived pain, while another group received the painful procedure first without the use of VR and then had another session of the procedure with VR. The latter group reported a greater pain reduction compared to the former. Two out of the 9 selected articles found no differences in pain perception between VR and standard cares, while another study investigated the VR efficacy on phlebotomy through 3 study groups and found out that VR efficacy was the same for external cold and vibration intervention but still more efficient than standard care [6, 9, 11]. We analyzed the pain reduction by comparing the indicated values with and without VR, with conflicting results. These studies reported a pain reduction of: 4/10 [5], 2.57/5 [10], 1.78 and 1.39 [8], 2.89 ± 0.2/10 [7], 3/10 [6], 1.15 ± 0.28/5 [12], 2.37/10 [13], and other didn’t show statistical differences [9, 11].

Despite the various methods of carrying out the studies, VR distraction showed a statistically significant reduction in pain. The sample of subjects was between 30 and 252 subjects, while the age range of 4–20 years seems to mark a cut-off for the use of VR in children aged 4 years. These findings are consistent with preliminary results from a pilot study currently being conducted by our research group. The study aims to evaluate the effects of VR on anxiety and pain during the execution of venous sampling in pediatric patients with cystic fibrosis and with the use of the Numerical Rating Scale pain and the State Trait Anxiety Inventory. From a preliminary data analysis, the results show significant pain, anxiety and stress reduction in subjects treated with VR during the venipuncture procedure.

## Conclusion

In conclusion, VR seems to be an effective tool for non-pharmacological pain reduction. Although there are few publications on this topic, they show that VR could be useful for patients who are forced to feel pain. Since in a few cases VR does not reduce pain compared to standard cares, pain reduction is the same in both VR and non-VR treatments. Moreover, the interaction between virtual exposure and opioids is not clear yet. Similarly, it is not clear if, for greater pain reduction, it is better to take therapy with VR first and then without or vice-versa. Although VR is an effective tool for pain reduction, most of the studies investigate its effect on acute pain only. For this reason, more studies are needed to better understand the effect of VR in the pediatric population, both on acute and chronic pain.

## Data Availability

All data generated or analyzed during this study are included in this published article.

## Abbreviations

ET: Exposure Therapy
MCDAS: Modified Child Dental Anxiety Scale
NRS: Numerical Rating Scale
PTSD: Post traumatic Stress Disorder
STAI: State Trait Anxiety Inventory;
VAS: Visual Analogue Scale
VR: Virtual Reality
VRET: Virtual Reality Exposure Therapy
WGRS: Word Graphic Rating Scale
FLACC: Face Legs Activity Cry Consolability
VOE: Vaso-Occlusive Episodes
SCD: Sickle Cell Disease
IAN: Inferior alveolar nerve block
